# Establishment and characterization of three new human breast cancer cell lines derived from Chinese breast cancer tissues

**DOI:** 10.1186/1475-2867-9-2

**Published:** 2009-01-02

**Authors:** Chao Shen, Meijia Gu, Dan Liang, Lixia Miao, Liu Hu, Congyi Zheng, Jiakuan Chen

**Affiliations:** 1 State Key Laboratory of Virology, College of Life Sciences, Wuhan University, Wuhan 430072, PR China; 2 China Center for Type Culture Collection, Wuhan University, Wuhan 430072, PR China; 3 Zhongnan Hospital, Wuhan University, Wuhan 430072, PR China

## Abstract

**Background:**

Breast cancer is a major malignancy affecting females worldwide. It is the most common cause of death from cancer in women. Cell lines are widely used in laboratory research and particularly as *in vitro *models in cancer research. But we found that the routinely used breast cancer cell lines were mostly derived from Caucasians or African-Americans. There were few standard models to study the pathogenic mechanism at molecular level and cell signaling pathway of breast cancer for Asian patients. It is quite necessary to establish new breast cancer cell lines from xanthoderm to study the pathogenic mechanism and therapeutic methods.

**Results:**

Three new breast cancer cell lines, designated BC-019, BC-020 and BC-021, were successfully established and characterized from breast invasive ductal carcinoma tissues of three Chinese female patients. These new cell lines growing as adherent monolayer with characteristic epithelial morphology could be maintained continuously *in vitro*, and they were ER-, PR- and C-erbB-2-positive. Their chromosomes showed high hyperdiploidy and complex rearrangements, and they displayed aggressive tumorigencity in tumorigenesis test.

**Conclusion:**

The three newly established breast cancer cell lines from Chinese patients were tested for a number of, and the results indicate that the cell lines were in good quality and could be served as new cell models in breast cancer study.

## Background

Breast cancer is a major malignancy affecting females worldwide. It is the most common cause of death from cancer in women [1-3]. Cell lines are widely used in laboratory research and particularly as *in vitro *models in cancer research. Apart from MCF-7, the most commonly used breast cancer cell line in the world derived from a pleural effusion in the Michigan Cancer Foundation [4], a number of other cell lines are routinely used as breast cancer models, such as BT20, MDA-MB-231, MDA-MB-435s, and T-47D [5]. Moreover, most *in vitro *studies using breast cancer cells are based on a few well-characterized cell lines, such as MCF-7, ZR-75-30, T-47D and MDA-MB-231, which have been established in culture for over 30 years[4,6,7]. Furthermore, most of these long established breast cancer cell lines are not derived from primary breast tumors, but from tumor metastases, especially aspirates or pleural effusions.

We also found that these routinely used breast cancer cell lines were mostly derived from Caucasians or African-Americans, such as MCF-7, ZR-75-30, T-47D and MDA-MB-468, but very rarely cell lines were derived from xanthoderm, particularly from Asians. In American Type Culture Collection (ATCC), there were more than 70 breast cancer cell lines, but only one of them was derived from Asian(Hs 739.T, ATCC NO.: CRL-7477) and one from East Indian (HCC1954, ATCC NO.: CRL-2338). And there were few standard models to study the pathogenic mechanism at molecular level and cell signaling pathway of breast cancer for Asian patients. Even in some studies on tumor pathologic features in Chinese patients, only the cell lines derived from Caucasians or African-Americans were used, so there were few experimental systems to study the pathogenic mechanism of breast cancer for Asian patients [8]. In our previous work about the breast cancer associated gene BRCA1, we also used the cell lines ZR-75-30 and MDA-MB-435S as investigate objects[9]. In China, the incidence rate of breast cancer has been seen increasing significantly both in urban and rural areas in the last three decades[10,11]. So it is quite necessary to establish new breast cancer cell lines from xanthoderm to study the pathogenic mechanism and therapeutic methods.

In this report, we established and characterized three new breast cancer cell lines BC-019, BC-020 and BC-021 from three Chinese patients. These cell lines which are positive for estrogen receptor (ER), progesterone receptor (PR) and C-erbB-2, could provide us with new experimental materials to study the pathogenic mechanism and to screen for new therapeutic reagents against breast cancer.

## Methods

### Primary Culture and Establishment of Cell Lines

Three breast cancer cell lines were established from three Chinese female breast cancer patients who underwent mammary gland excision in Zhongnan Hospital of Wuhan University. The study was approved by the hospital ethics committee and patient's informed consent was obtained. The tumor specimen from primary mammary glands was removed at the excision operation. Standard blocks were then taken for pathological examination (Table 1). Tissue samples were processed for primary culture as described previously [12]. Briefly, tumor specimen was washed five times in Hanks' balanced saline and minced with scissors into small pieces of 1–3 mm^3^. The small tissue fragments were placed in three 25-cm^2 ^culture flasks and cultured at 37°C in a humidified incubator containing 5% carbon dioxide in Eagle's minimum essential medium (MEM) with non-essential amino acids supplemented with 10% fetal bovine serum (FBS, Invitrogen), penicillin (100 U/mL), and streptomycin (100 μg/mL). Half of the medium was replaced every 3–4 days. Sparsely outgrowth colonies of polygonal epithelial cells from the tissue pieces were observed within 5 days. On the 12th day after initiation of the primary culture, the epithelial cells were transferred selectively to fresh culture flasks. Cells were harvested after treatment with 0.25% trypsin and 0.02% EDTA solution and subcultured with a 1:3 split ratio. During the subsequent period of continuous propagation by subculture, the cells were sampled at intervals, resuspended in a freezing medium (80% MEM, 10% FBS, and 10% dimethyl sulfoxide [DMSO]), and stored in liquid nitrogen every ten passages. After quickly thawing at 37°C, the frozen cells could be propagated in culture without noticeable change in growth and morphology. The cell lines were named BC (breast cancer)-019, BC-020, BC-021 and deposited in China Center for Type Culture Collection (CCTCC) with designation number CCTCC-GDC0198, CCTCC-GDC0199 and CCTCC-GDC0200, respectively.

**Table 1:** Origins of the three breast cancer cell lines

**Cell line**	**Species**	**Gender**	**Age**	**Source**	**Pathology**
BC-019	Human, Chinese	Female	47	Right breast	Invasive ductal carcinoma
BC-020	Human, Chinese	Female	50	Left breast	Invasive ductal carcinoma
BC-021	Human, Chinese	Female	42	Left breast	Invasive ductal carcinoma

### Assay of Growth Characteristics of Cell Line

BC-019, BC-020 and BC-021 cells at the same passage 21 (P21) were used to plot the growth curve by using the 3-(4,5-dimethylthiazol-2-yl)-2,5-diphenyl tetrazolium bromide (MTT) method [13-16]. The cells were plated into 96 well plates, 2 × 10^3 ^cells per well and 7 plates per cell line. One plate of each cell line was assayed every 24 hours. The absorbance was measured in a microtiter plate reader at 570 nm as the test wavelength and 690 nm as the reference wavelength. The number of cells was calculated by the result of MTT compared with the cell number at the first day. The growth curves were plotted and the population doubling time of these 3 cell lines were calculated during the exponential growth phase of the cells.

### Contamination Tests

#### Testing for Bacteria and Fungi

Detection of the contaminants in cell lines followed the standard procedures of American Type Culture Collection (ATCC)[17]. The cells at passage 21 (P21) were cultured with the above-mentioned medium without the addition of antibiotics for 5 days. Eight microbial media were used for the detection of bacteria and fungi, including thioglycollate medium (Difco 0256-01), Sabouraud dextrose broth (Difco 0382-01), Trypticase soy broth (BBL 01-162), Brain heart infusion (BHI) broth (Difco 0037-01-6), YM broth (Difco 0711-01), Blood agar plates (with fresh defibrinated rabbit blood) (Difco 0045-01), Nutrient broth incline planes (Difco 003-01) and Martin modified medium. Of the eight media employed, trypticase soy, BHI, blood agar and thioglycollate were used to detect a wide range of bacterial contaminants. Sabouraud broth, YM broth, nutrient broth incline planes and Martin modified medium were used to detect fungal contaminants. The test regimen was listed in Table 2. 1 ml of the 5-day cultured supernatant was added into each medium, and incubated at different temperatures for 14 and 21 days. Each medium at each temperature was tested in triplicate.

**Table 2:** Regimen for detecting bacterial and fugal contamination

Test medium	Temperature (°C)	Gas phase	Observation time (days)
Blood agar plates	37 37	Aerobic Anaerobic	14
Brain heart infusion broth	37 26	Aerobic	14
Trypticase soy broth	37 26	Aerobic	14
Thioglycollate broth	37 26	Aerobic	14
Sabouraud broth	37 26	Aerobic	21
YM broth	37 26	Aerobic	21
Nutrient broth incline planes	37 26	Aerobic	21
Martin modified medium	37 26	Aerobic	21

#### Testing for Mycoplasma

The cell lines were examined for mycoplasma using both direct and indirect assays [17]. In the direct assays, mycoplasma growth agar plates were prepared with mycoplasma agar base (Becton-Dickinson 11456) and horse serum (GIBCO). 1.0 ml of the 5-day cultured supernatant was inoculated onto the mycoplasma growth agar plates and incubated anaerobically at 37°C. Vero (CCTCC GDC0029) and B6yH4 (CCTCC GDC0017) cells obtained from CCTCC were used as negative and positive control respectively. Typical mycoplasma colonies were microscopically examined very week for at least 3 weeks.

In the indirect assay, mycoplasma DNA was examined by staining the cells with Hoechst 33258. Vero cell line was used as the indicator cell in the DNA staining procedure. Vero cells were inoculated on a cover slip in 12-well microplates with 1.0 × 10^5 ^cells/well. After 24 hours, 0.2 ml to 0.5 ml of each test cell line were added to the wells of Vero cells, 2 wells/cell line. 0.5 ml supernatant of B6yH4 cells was added to 2 wells as positive control and 1.0 ml culture medium was added to 2 wells as negative control. The 12-well microplates were incubated at 37°C in a humidified incubator containing 5% carbon dioxide. After 6 days, cells were washed with PBS twice, fixed with fixative (methanol: acetic acid = 3:1) for 15 minutes twice and stained with Hoechst 33258 in the dark for 45 minutes. The cover slips were then washed with distilled water three times, dried in the dark at room temperature, placed in a drop of mounting medium (10% glycerin in PBS) on glass slides, and observed under fluorescence microscope with 330–380 nm excitation filter. Extracellular fluorescence indicated mycolpasma contamination.

### Isoenzyme Analysis

To verify that the three breast cancer cell lines were derived from humans without cross-contamination, we compared the profiles of lactate dehydrogenase (LD) and malate dehydrogenase (MD) from the 3 breast cancer cell lines. HeLa cell line and mouse fibroblast L929 cell line were used as standard references. LD and MD were analyzed using AuthentiKit™ (Innovative Chemistry Marshfield, MA) following the manufacturer instructions.

### Immunocytochemistry Analysis

ER, PR and C-erbB-2 had been used as marker for the detection of breast cancer in clinical diagnosis [18-21]. The expression of ER, PR and C-erbB-2 were detected by using SuperRmEPC™ Breast Cancer detection Kit from Maxim Biotechnology Development Co., Ltd MRC-5 cell line (ATCC CCL-171) and MCF-7 cell line (ATCC HTB-22) were used as negative control and positive control, respectively.

### Assay of Tumorigenicity in Nude Mice in vivo

Large-scale cultures were prepared at P21 for BC-019, P22 for BC-020 and P20 for BC-021. Cells were washed twice with MEM without serum and antibiotics and adjusted to 5~10 × 10^7 ^cells/ml in PBS. The same procedure was done to MRC-5 cell line and MCF-7 cell line, the negative and positive controls. MRC-5 and MCF-7 were cultured in MEM supplemented with 10% fetal bovine serum, penicillin (100 U/mL), and streptomycin (100 μg/mL). 10 μg/mL bovine insulin was added to the medium for MCF-7. 4 weeks old nude mice were injected subcutaneously with 0.2 ml cell suspension at left groin. Each cell line was used to inject 10 mice. All the mice were examined within 8 weeks [22-24].

### Chromosomal Analysis

BC-019, BC-020 and BC-021 cells at passage 23 were used for chromosome analysis. Metaphase cells were collected after 3-hour colcemid (0.2 μg/mL) exposure and incubation in 0.075 M KCl solution at 37°C for 40 min. After fixing with a mixture of methanol and glacial acetic acid (3:1, v/v), cell suspensions were spread onto cold slides. The slides were stained with 3% Giemsa for morphological examination and chromosomes analysis [25]. G-banding of the chromosomes was obtained by carried out by standard trypsin-Giemsa banding technique[26,27].

## Results

### Establishment of Cell Lines

**Figure 1:**
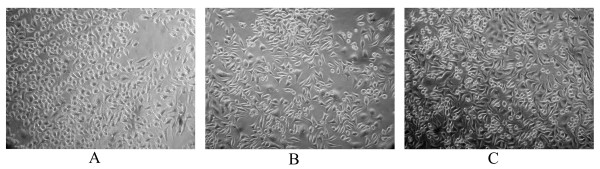
**Morphology of three breast cancer cell lines**. A: BC-019 cell line at passage 21 (×100); B: BC-020 cell line at passage 22 (×100); C: BC-021 cells at passage 20 (×100).

The epithelial cells grew gradually from explants in all the 25-cm^2 ^culture flasks and formed sparse colonies within 5 days of primary culture. The first successful subculture of 3 cell lines was performed on about 14th day and the second one 3 days later. 5 passages later, cells were subcultured at a split ratio of 1:3 every 3–4 days. The breast cancer cells grew as adherent monolayer with characteristic epithelial morphological features (Fig. 1). The breast cancer cells have grown continuously for over 6 months after initiation and have undergone more than 45 passages (BC-019: 51 passages; BC-020: 46 passages; BC-021: 48 passages). After thawing from liquid nitrogen, the cryopreserved cells could be propagated in culture without noticeable change in growth and morphology.

### Growth Characteristics of Cell Lines

**Figure 2:**
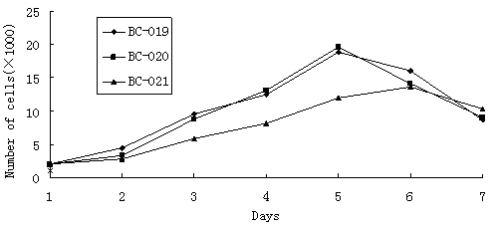
**Growth curves of 3 breast cancer cell lines**. The cells seeded at 2 × 10^3 ^cells per well and measured by MTT every 24 h. The growth curves were plotted with the cells numbers within 7 days.

The growth kinetics of BC-019, BC-020 and BC-021 cells at passage 21 were studied. The growth curves of these cell lines were shown in Fig. 2. The population-doubling time of BC-019, BC-020 and BC-021 were 36 hours, 35 hours and 46 hours, respectively.

### Contamination Tests

**Figure 3:**
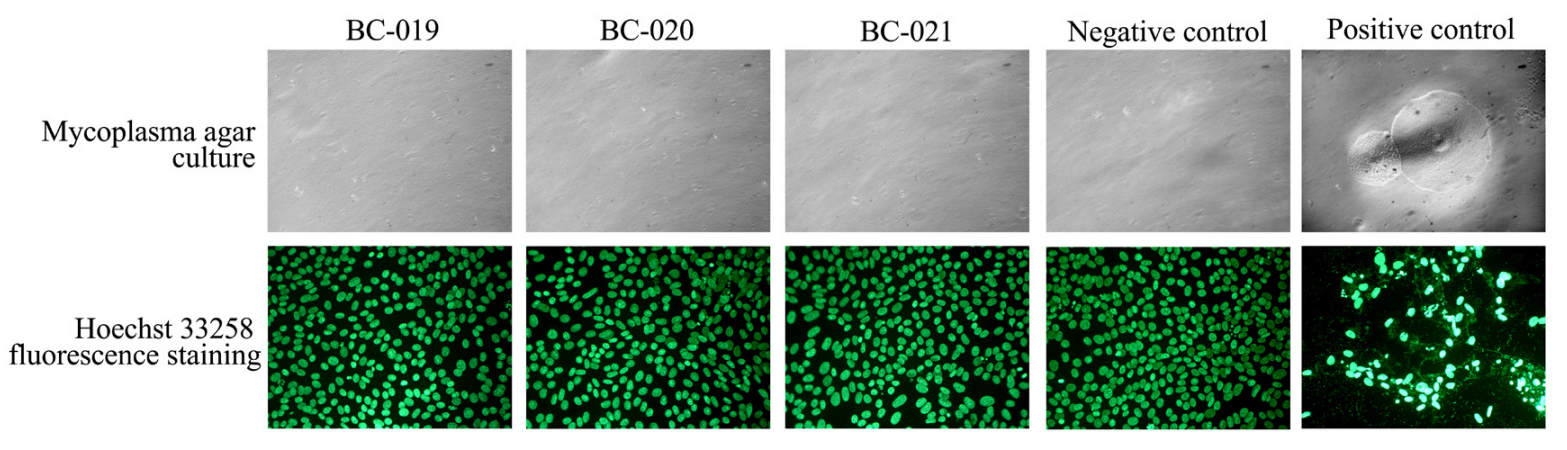
**Mycoplasma detection of breast cancer cell lines**. Mycoplasma colonies were seen as fried-egg morphology at the positive control agar plates Mycoplasma contamination was seen as the numerous small extracellular fluorescing particles by Hoechst 33258 fluorescence staining.

These test regimens could detect most common bacterial, fungal organisms and mycoplasma in cell cultures. We did not detect bacterial and fungal contaminants in the three breast cancer cell lines by using eight growth media. Mycoplasma agar culture and Hoechst 33258 fluorescence staining indicated that there was no mycoplasma in the three new breast cancer cell lines (Fig. 3). Altogether, contamination tests indicated that the three breast cancer cell lines were free of bacterial and fungal agents as well as mycoplasma.

### Isoenzyme Analysis

**Figure 4:**
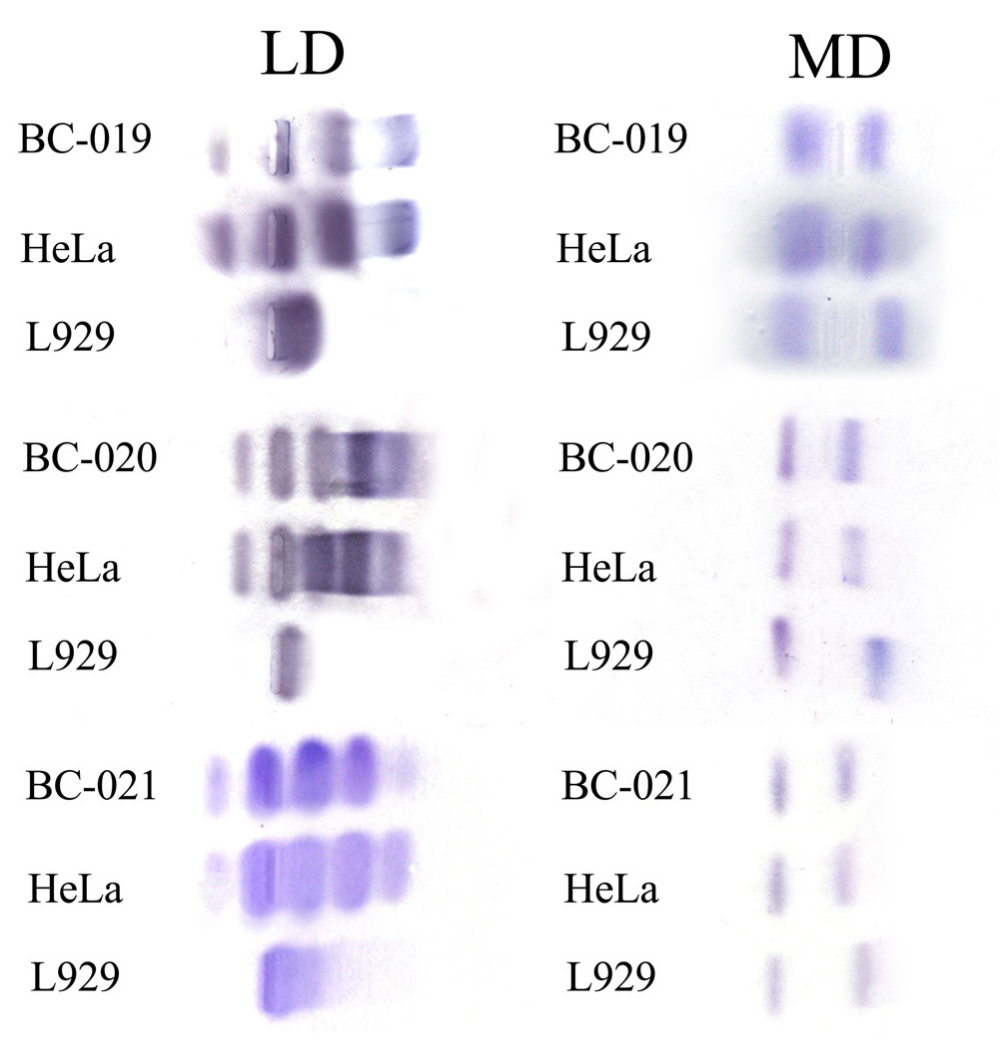
**Isoenzymology analysis of lactate dehydrogenase (LD) and malate dehydrogenase (MD) in the three breast cancer cell lines**. HeLa is human control and L929 is mouse control. The bands of breast cancer cells were identical to those from HeLa but distinct from L929, indicating that the breast cancer cells had the same origin as of HeLa cells.

We compared the profiles of lactate dehydrogenase and malate dehydrogenase from the three breast cancer cell lines together with HeLa cells and mouse fibroblast L929 cells. The results were shown in Fig. 4. The profiles of the two dehydrogenases from these breast cancer cell lines were identical to those from HeLa cells, but distinct from those of mouse fibroblast cell line L929. These data showed that the origin of these three cell lines was human tissue.

### The Expression of ER, PR and C-erbB-2 MarKers

**Figure 5:**
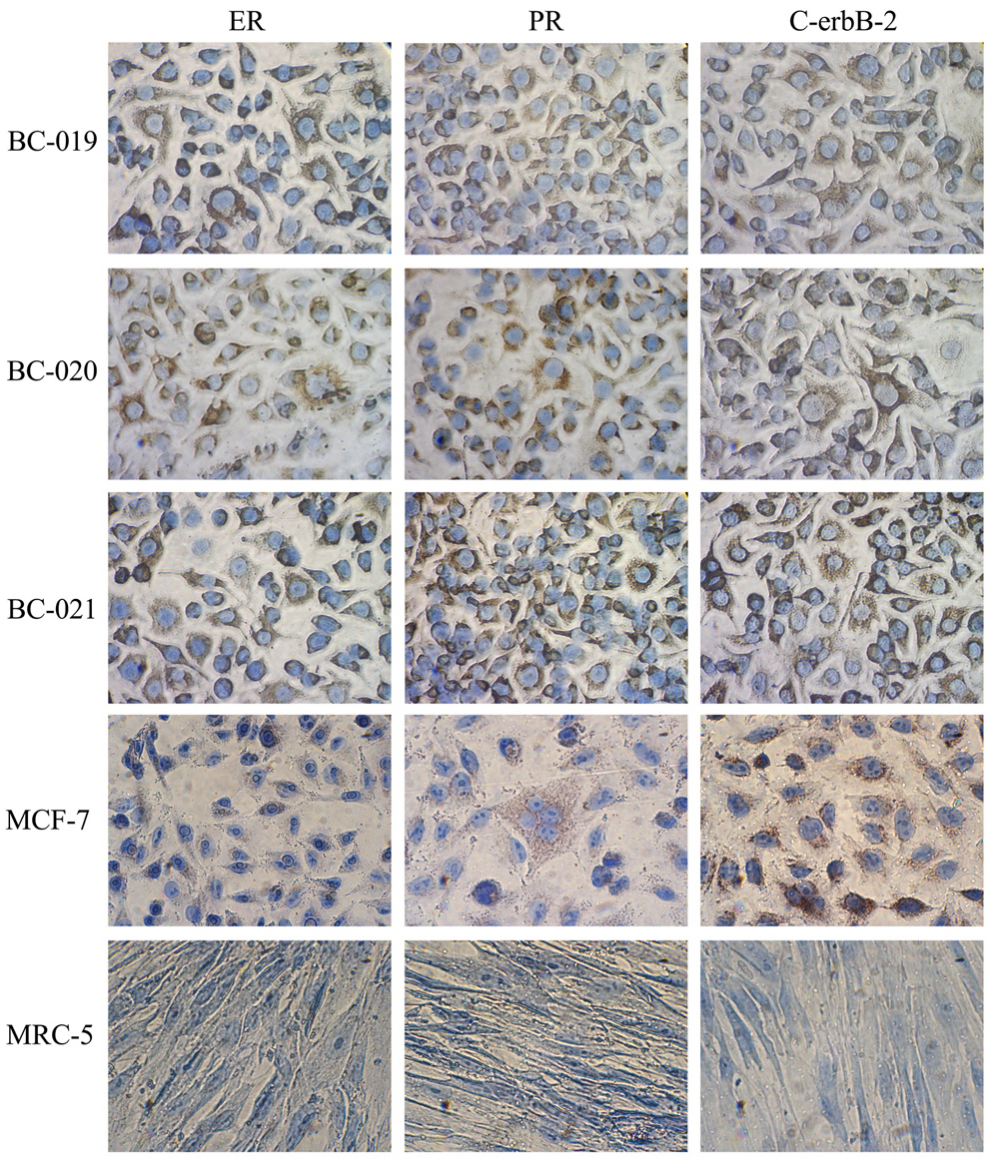
**Immunocytochemical analysis of three breast cancer cell lines**. The expression of ER, PR and C-erbB-2 were detected by using SuperRmEPC™ Breast Cancer detection Kit, all the three cell lines were positive for the three markers, in immunocytochemistry anslysis.

The expression of ER, PR and C-erbB-2 in three breast cancer cell lines was detected by using the SuperRmEPC™ Breast Cancer detection Kit. As shown in Fig. 5, all the three new breast cancer cell lines were positive for ER, PR and C-erbB-2 and there were no notable difference at the expression level among the three breast cancer cell lines. Compared with MCF-7, the expression level of ER and PR in breast cancer cell lines was higher than that of MCF-7 and the expression of C-erbB-2 was similar with that of MCF-7. On the other hand, the negative control cell line MRC-5 did not appear to express ER, PR and C-erbB-2. Altogether, the data of immunocytochemistry analysis provided the evidence that the three newly established cancer cell lines are from human breast malignancy.

### Highly Tumorigenicity of Three Cell Lines in Nude Mice

**Figure 6:**
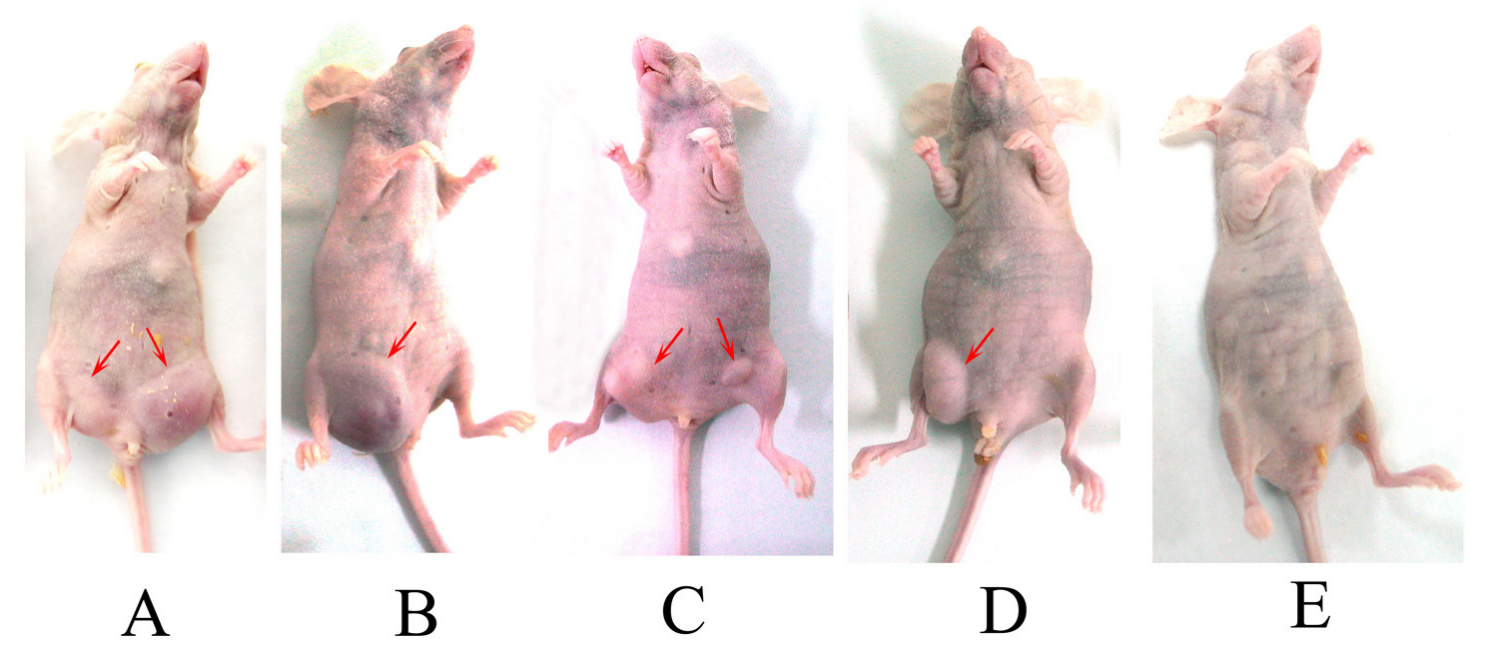
**Tumorigenicity test of breast cancer cells in nude mice**. Red arrows indicated the tumors in the nude mice. A: One nude mice 30 days post-injection of BC-019 cells, where tumors appeared on both groins; B: One nude mice 30 days post-injection of BC-020 cells; C: One nude mice 30 days post-injection of BC-021 cells, where tumors appeared on both groins; D: One nude mice 30 days post-injection of MCF-7 cells (positive control); E: One nude mice 30 days post-injection of MRC-5 cells (negative control).

To further investigate the *in vivo *tumorigenicity of these new breast cancer cell lines, the xenograft transplanation to nude mice was performed. Within 10–14 days after subcutaneous injection of breast cancer cells, visible subcutaneous tumors were developed in all of the 30 nude mice at the site of inoculation while the tumors in MCF-7-injected nude mice appeared within 20 days post-injection. The dimension of these tumors ranged from 1.5 to 2.0 cm within 5 weeks post-injection (Fig. 6). At the same time, the negative control cell line MRC-5 did not exhibit tumorigenicity in 10 nude mice (Fig. 6E). Furthermore, tumor metastasis occurred occasionally in the nude mice injected with BC-019 cells (1/10) and BC-021 cells (1/10)(Fig. 6-A, C). Tumors appeared on both sides of the groins, while the cells were only injected into the left groin. No metastasis was found in the nude mice injected with either BC-020 cells or MCF-7 cells. This result indicated that the newly established breast cancer cell lines were highly tumorigenic *in vivo*.

### Chromosome Analysis

**Figure 7:**
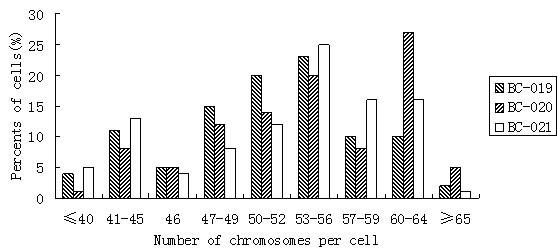
**Distribution of chromosome numbers in three breast cancer cell lines**.

Karyotype analyses were carried out to determine the modal number of chromosomes by standard Giemsa staining. 1000 cells of each cell line were counted and chromosome numbers in BC-019, BC-020 and BC-021 cells were ranged between 35 to 67, 40 to 70 and 38 to 65 with median ranges 53–56, 53–56 and 60–64 respectively (Fig. 7). In all the three cell lines, the karyotypes were mostly multiploid and many chromosomal abnormalities were observed. Many chromosomes were fractured at the centromeres and most of the fractured chromosomes could not be identified. The chromosomal abnormalities were much more severe in BC-021 cell line.

## Discussion

Well-characterized human cancer cell lines are important resources for studying cancer cell biology, as well as for developing new strategies against cancer cell growth and progression[28]. In this study, we reported the establishment and characterization of three new breast cancer cell lines, BC-019, BC-020 and BC-021, which were derived from breast invasive ductal carcinoma tissues of Chinese female patients.

The newly established cells grew as adherent monolayer with characteristic epithelial morphology. The cultured cells maintained consistent morphology from the primary culture to the subsequent subculture passages. These cells have grown continuously for over 6 months and have undergone more than 45 passages. They appeared to be permanent cell lines since growth continued after recovery from cryopreservation. The population-doubling time of BC-019, BC-020 and BC-021 were 36 hours, 35 hours and 46 hours, respectively.

We did not add any insulin to the medium in primary culture to maintain the cell lines, while it was necessary for most of routinely used breast cancer cell lines, such as MCF-7, T-47D, MDA-MB-435S and so on[4,7,29]. We found that the absence of insulin in the culture medium did not affect the characteristics of these three cell lines. Therefore, it is more convenient to culture these cell lines.

The contaminations would do great harm to cell cultures. In our contamination tests, no bacteria and fungi or mycoplasma could be found in the three cell lines. Isoenzyme analysis was used to verify the origin of species. We analyzed the lactate dehydrogenase and malate dehydrogenase of the new cell lines. The results indicated that the origin of the three cell lines was identical to HeLa cell line. So the three breast cancer cell lines were derived from human tissues and there were no other species cross-contamination during the proliferation. Meanwhile, all the three cell lines were positive for the expression of estrogen receptor, progesterone receptor and C-erbB-2. This result demonstrated clearly that the three cell lines were derived from human breast cancer tissues.

The karyotypes of the three breast cancer cell lines showed chromosomes with hyperdiploidy and complex rearrangements. The pronounced heterogeneity suggested that these three cell lines consisted of karyotypically different clones. The main heterogeneity of the three cell lines was chromosome fracture at the centromeres and most of the fractured chromosomes could not be identified. The fractured chromosomes were in larger number in BC-021 than in the other two cell lines. The hyperdiploidy (> 50 chromosomes) may arise from fracture of the chromosomes.

The three breast cancer cell lines had been proved to have severe tumorigenicity when injected into nude mice, and their tumorigenicity was higher than that of MCF-7 cell line. Metastasis could be occasionally observed in nude mice injected with BC-019 cells and BC-021 cells, but not in mice injected with BC-020 cells and MCF-7 cells. It was well known that breast cancer cell lines tend to be less tumorigenic and less metastatic than other cell lines derived from lung, renal and colon carcinoma when injected subcutaneously in nude mice[30]. Interestingly, these three cell lines could form tumors at 100% rate when injected in nude mice within two weeks, and the maximum dimension of these tumors reached 2.0 cm within 5 weeks post-injection. The size of the tumor was bigger than that formed by MCF-7 under same conditions. It seems that these newly established breast cancer cell lines could be new *in vivo *models for studying the biology of breast cancer.

Most of the previously established breast cancer cell lines used in research did not derive from primary breast tumors, but from tumor metastases, especially ascites or pleural effusions. While primary cultures are indispensable for direct comparison to the tissue of origin, immortalized cell lines are necessary for long-term studies [31,32]. Therefore, these new cell lines will be valuable tools in breast cancer research. Not only are cells directly isolated from the tumor site, but also detailed pathology is available to allow the characteristics of the culture to be compared with those of the original tumor.

Moreover, most of the published breast cancer cell lines were derived from Caucasians, which limited the study of pathogenic mechanism of breast cancer among Asian patients. To resolve this problem, we established three breast cancer cell lines which were derived from Chinese and provided a research resource and tool to compare the difference of cells and tumors between the two races.

## Conclusion

We established and characterized three new human breast cancer cell lines designating BC-019, BC-020 and BC-021 from three Chinese patients. These three cell lines would provide us new experimental models to study the pathogenic mechanism, to investigate the biological behavior and to test new therapeutic reagents for breast cancer.

## Abbreviations

ATCC: American Type Culture Collection; CCTCC: China Center for Type Culture Collection.

## Competing interests

The authors declare that they have no competing interests.

## Authors' contributions

CS, MG, DL, LM and LH carried out all experimental work. CS and CZ conceived ideas, analyzed data and wrote manuscript. JC contributed in collecting tissues of the patients and pathology assay. CS, LH and CZ analyzed results and evaluated manuscript. All authors read and approved the final manuscript.
